# Liver transplantation for a patient with Turner syndrome presenting severe portal hypertension: a case report and literature review

**DOI:** 10.1186/s40792-016-0194-x

**Published:** 2016-06-29

**Authors:** Seiichi Kawabata, Seisuke Sakamoto, Masaki Honda, Shintaro Hayashida, Hidekazu Yamamoto, Yoshiki Mikami, Yukihiro Inomata

**Affiliations:** Department of Transplantation and Pediatric Surgery, Kumamoto University, 1-1-1, Honjo, Chuo-ku, Kumamoto 862-8556 Japan; Department of Pathology, Kumamoto University, 1-1-1, Honjo, Chuo-ku, Kumamoto 862-8556 Japan; Division of Transplant Surgery, Organ Transplantation Center, National Center for Child Health and Development, 2-10-1, Okura, Setagaya-ku, Tokyo 157-8535 Japan

**Keywords:** Liver transplantation, Obliterative portal venopathy, Turner syndrome

## Abstract

**Background:**

Liver involvement in Turner syndrome (TS) patients has been more clearly clarified in recent years. Most of the clinical manifestations in TS are asymptomatic and can be detected as liver test abnormalities; however, a few cases may present with end-stage liver disease and thus require liver transplantation (LT). To the best of our knowledge, only three cases undergoing LT for liver involvements in TS have been previously reported.

**Case presentation:**

A 30-year-old female successfully underwent living donor LT for liver dysfunction related to TS syndrome. The diagnosis of TS was established by a cytogenetic analysis at 16 years of age. She received several sessions of endoscopic therapy for recurrent esophageal varices, which was complicated by ascites and spontaneous bacterial peritonitis since 27 years of age. Radiological findings of her liver before LT chronologically showed the progression of atrophy with disturbance of the major portal inflow. And then, she was finally indicated for LT. Pathologic findings of the explanted liver showed vascular abnormalities, obliterative portal venopathy, which may have induced liver dysfunction with severe portal hypertension. The patient’s postoperative course was uneventful.

**Conclusions:**

The clinicopathologic information obtained by the current case can provide an insight into understanding pathophysiological mechanisms of liver involvement in TS patients. TS patients presenting with severe liver atrophy and disturbance of the major portal inflow should be indicated for LT.

## Background

Turner syndrome (TS), resulting from a total or partial loss of the X chromosome, is characterized by a short stature, ovarian dysgenesis, dysmorphic syndrome, cardiovascular, and renal malformations [1]. Liver involvement in this syndrome is mostly asymptomatic and detected by systematic blood testing [2]. The prevalence of liver function abnormalities ranges from 20 to 80 % [3, 4], and a few reports have described severe consequences of the liver involvement [5]. The risk of cirrhosis in patients with TS appears to be approximately fivefold higher compared with a control group of patients [6]. However, liver transplantation (LT) in cases of TS has been rarely reported [7–9], and thus the microscopic features of liver abnormalities associated with this condition remains to be elucidated.

We herein report a case of LT indicated for severe portal hypertension associated with TS and describe the histological findings of the explanted liver, which can provide an insight into understanding the pathophysiological state of liver involvement in TS patients.

## Case presentation

**Fig. 1:**
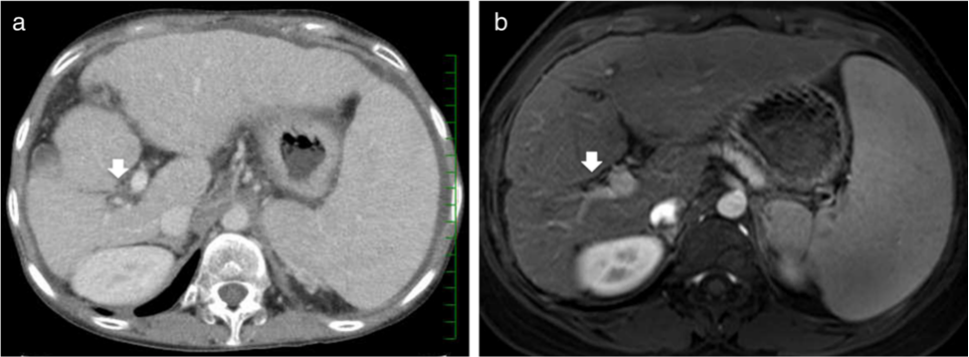
Chronological changes in radiologic findings of the liver. Enhanced abdominal computed tomography just before LDLT (**a**) and enhanced abdominal magnetic resonance imaging at 25 years of age (**b**). The progression of atrophy of the right lobe and severe stricture of the right portal vein (*white arrow*) is revealed

A 30-year-old female with TS, presenting with liver dysfunction, was referred to the hospital for consideration of LT. Her height and weight were 149 cm and 40 kg, respectively. At 16 years of age, she was concerned about her short stature, and the diagnosis of TS was established by a cytogenetic analysis, which showed a mosaic pattern of 45, X and 46, X, r(X). Thereafter, she had received growth hormone therapy until 18 years of age. Liver test abnormalities were noted at this time. A microscopic examination of a liver needle biopsy specimen demonstrated grade 3 fibrosis according to the Metavir classification. She was monitored with estrogen/progesterone therapy since 24 years of age, and never showed any symptoms related to liver dysfunction until she received endoscopic therapy for esophageal varices at 27 years of age. She received several sessions of endoscopic therapy for recurrent esophageal varices, which was complicated by ascites and spontaneous bacterial peritonitis, necessitating frequent hospitalization. She never experienced any episodes of encephalopathy and hyperammonemia. Although the elevation of liver enzymes was mild, she presented with severe pancytopenia and hypoalbuminemia (Table 1). Enhanced abdominal computed tomography revealed the progression of atrophy of the right lobe and severe stricture of the right portal vein (Fig. 1a) compared to the liver findings using enhanced abdominal magnetic resonance imaging at 25 years of age (Fig. 1b). Therefore, living donor LT (LDLT) was indicated as a curative therapeutic option. Her mother (52 years of age) voluntarily donated her left lobe weighing 410 g. Rituximab at a dose of 375 mg/m^3^ body surface area was used 2 weeks prior to the patient’s scheduled LDLT, and concomitant splenectomy was performed at the time of LDLT for prophylaxis for antibody-mediated rejection due to ABO-blood type incompatibility between the mother and daughter. The patient’s native liver revealed atrophic right lobe and nearly complete obstruction of right portal vein. However, the consistency of the liver appeared to be less firm than expected. The structure from the portal main trunk to left portal vein was moderately dilated without any wall abnormalities. Vascular reconstructions were performed using the main portal vein for portal vein reconstruction, while biliary reconstruction was performed using hepaticojejunostomy. The patient’s postoperative course was uneventful, and she was discharged without any surgical complications 1 month after LDLT.

**Table 1:** Chronological changes in laboratory test results

	At first biopsy (17 years of age)	At first episode of esophageal varices (27 years of age)	At LDLT (30 years of age)
AST (U/L)	38	65	51
ALT (U/L)	42	90	36
GGTP (U/L)	40	74	69
T-Bil (mg/dL)	1.6	3.4	2.5
D-Bil (mg/dL)	0.4	0.3	0.4
ALB (g/dL)	4.2	4.3	2.8
WBC (×10^3^/μL)	4.5	1.7	1.9
PLT (×10^3^/μL)	9.7	48	62
PT-INR	1.05	1.15	1.06

*ALB* albumen, *ALT* alanine transaminase, *AST* aspartate transaminase, *D-Bil* direct bilirubin, *GGTP* gamma-glutamyl transferase, *LDLT* living donor liver transplantation, *PLT* platelet, *PT-INR* prothrombin time-international normalized ratio, *T-Bil* total bilirubin, *WBC* white blood cell

### Pathologic findings of the explanted liver

**Fig. 2:**
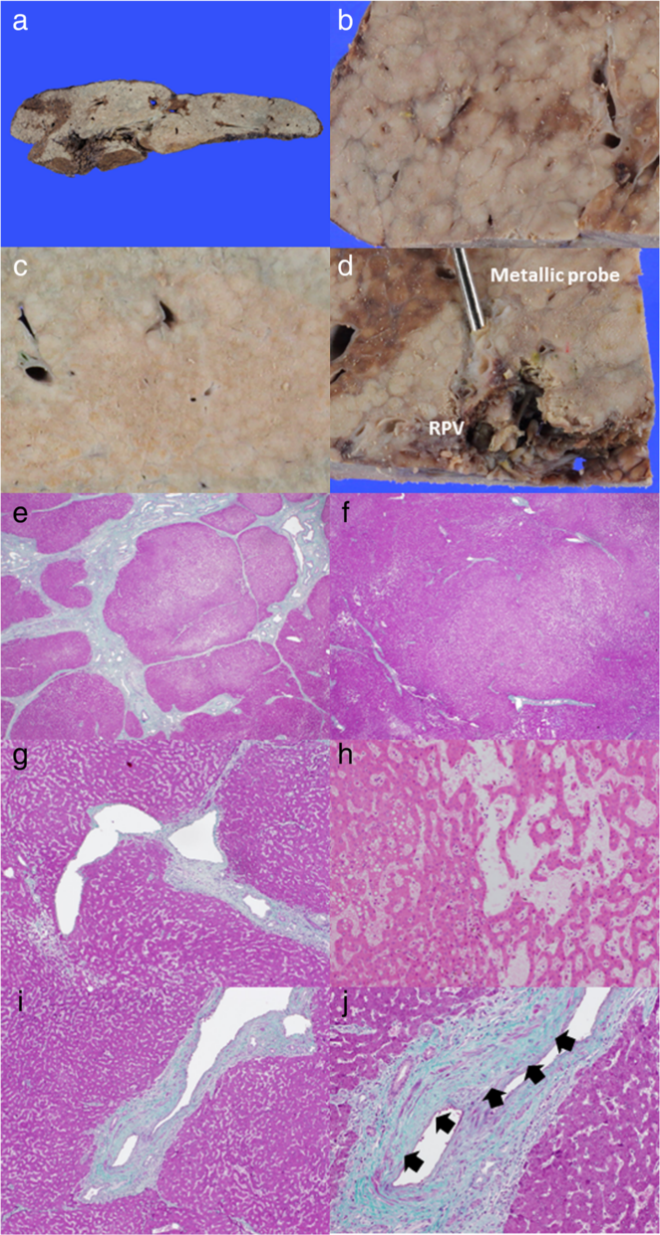
Pathologic findings of the explanted liver. The right lobe appears to be rather small compared with the left lobe (**a**). On the cut surface, vague nodularity is discernible on the right side (**b**), whereas nodularity is obscure on the left side (**c**). The right portal vein (RPV) became narrowed. Metallic probe indicates a narrowing site (**d**). A low-power view (×1.25 objective) showing nodules surrounded by a fibrous septum in the right lobe (**e** Masson’s trichrome stain), and a poorly demarcated and rather pale area of nodularity resulting from hyperplastic changes in hepatocytes with sinusoidal dilation, surrounded by incomplete fibrous bridging and atrophic areas of the hepatic parenchyma in the left lobe (**f** Masson’s trichrome stain). A dilated small-sized peripheral portal vein, protruding into the hepatic parenchyma (**g** Masson’s trichrome stain) and sinusoidal dilatation (**h** H&E stain), consistent with portal hypertension, is observed. Interlobular portal veins showing luminal narrowing and occasional dilatation, with intimal thickening (*black arrow*) (**i**, **j** Masson’s trichrome stain)

The explanted liver weighed 673 g. Grossly, the cut surface showed vague nodularity, particularly in the right lobe which showed significant atrophy (Fig. 2a, b). The left liver also showed nodularity, although it appeared to be obscure compared with the right side (Fig. 2c). The right portal vein became narrowed without thrombus (Fig. 2d), while the left portal venous system remained patent toward the periphery.

A microscopic examination revealed relatively well-demarcated nodules with a fibrous septum in the right lobe (Fig. 2d) and vague nodularity due to hyperplastic changes in hepatocytes with incomplete fibrous bridging in the left lobe (Fig. 2e). In addition, dilatation of a small-sized portal vein protruding into the hepatic parenchyma (Fig. 2f) and sinusoidal dilation (Fig. 2g) were also observed in areas of both lobes. Medium-sized interlobular portal veins showed various degrees of luminal narrowing and dilatation, with mild to moderate intimal thickening (Fig. 2h, i). There was heterogeneity in the thickness of the smooth muscle wall. Evidence of thromboembolism, including fenestration suggesting recurrent thrombosis, was not identified.

### Discussion

In the English-language literature, there are three cases of LT for liver involvements in TS [7–9]. The clinicopathologic features of these three cases and the current case are summarized in Table 2. In all cases, the patients were adults and the indications for LT were related to end-stage liver disease and/or portal hypertension. The histological findings of the explanted livers were typically vascular abnormalities unassociated with severe liver fibrosis and grossly appeared to be atrophic. One patient died of respiratory failure and fungal urosepsis with normal hepatic graft function [8].

**Table 2:** Literature review of liver transplantation for the cases with Turner syndrome

Case no.	Age at LT	Indication of LT	Type of LT	Histology of explanted liver	Outcome	Ref. no.
1	n/d	Uncontrolledvaricealbleeding, intractable cholestasis	OLT	Obliterative portal venopathy, periductal fibrosis, multiple focal nodular hyperplasia	n/d	7
2	52 years	Encephalopathy, gastrointestinal bleeding, refractory ascites/hydrothorax, hepatorenal syndrome	OLT	Atrophic liver, portal vein aneurysm and thrombus, small portal branches, no fibrosis	Died	8
3	44 years	Worsening liver function	OLT?	n/d	Alive	9
Current case	30 years	Variceal bleeding, refractory ascites, hypersplenism	LDLT	Obliterative portal venopathy, sinusoidal dilatation	Alive	–

*LDLT* living donor liver transplantation, *n/d* not described, *OLT* orthotopic liver transplantation

Liver abnormalities associated with TS hitherto described [2] include steatosis and steatohepatitis, which are relatively common in this syndrome, and are considered to be the result of overweight status, impaired insulin secretion, and diabetes mellitus [10]. Steatohepatitis may lead to fibrosis and the subsequent progression to cirrhosis. Another condition associated with TS represents liver architectural changes secondary to alteration of the hemodynamic state resulting from vascular abnormalities. Patients with TS occasionally may have nodular regenerative hyperplasia (NRH) or multiple focal nodular hyperplasia (FNH). The former is currently thought to represent a form of adaptation to microcirculatory disturbances causing heterogeneous distribution of the intrahepatic blood flow [11, 12], while the latter is also considered to be a focal hyperplastic hepatocellular response caused by focal deprivation of portal perfusion and enhanced arterial perfusion in the corresponding area [13]. Venous malformations, such as agenesia or hypoplasia of the portal venous system, may occur in TS [14]. A case of presinusoidal portal hypertension caused by congenital hypoplasia of the intrahepatic portal system, known as the Cruveilhier-Baumgarten disease, was reported [8]. Moreover, vascular abnormalities, especially cardiovascular malformations including bicuspid aortic valve and coarctation of the aorta, are common in TS [15]. Therefore, there is circumstantial evidence to suggest that vascular hepatic involvement in TS patients is part of a more general disorder, likely of congenital origin, involving vessels of different sizes, types, and locations [2, 7].

As mentioned above, previously described cases of LT for patients with TS are limited to three in the literature; however, some cases of major TS-related complications among patients awaiting LT have been reported [7, 9, 16]. According to the cohort study conducted by Roulot et al., portal hypertension was only observed in patients with marked architectural changes, consisting of NRH, FNH, and cirrhosis. Conversely, none of the patients without marked architectural changes experienced progressive or decompensated liver disease [7]. One explanted liver in their study revealed areas of multiple FNH coexisting with areas of sinusoidal dilatation and obliterative portal venopathy, while the other four patients with marked architectural changes showed changes corresponding to obliterative portal venopathy at biopsy. Obliterative portal venopathy can induce local hepatic hypoxia and atrophy, and it may be considered as one of the features indicating a poor prognosis. The laboratory and imaging features did not differentiate between patients with and without abnormal liver architecture in half of their patients; therefore, the authors recommended a liver biopsy for diagnostic and prognostic purposes in TS patients with persistently abnormal liver tests [7].

In the current case, imaging studies revealed sequential morphological changes toward obstruction of the right portal vein and atrophy of the right lobe. On the other hand, the left portal venous system was patent and the left lobe appeared with compensatory hypertrophy. Although our examination failed to reveal complete occlusion of the portal veins, dilatation with intimal thickening and occasional areas of thinning of the muscular wall suggested underlying obliterative portal venopathy, which may involve vessels of different sizes, types and locations, as previously described in some literature [2, 7]. Furthermore, the left lobe with a patent portal venous system might not have a reserve to compensate the liver function when the right lobe progressed to severe atrophy. Portosystemic shunting and splenectomy might have been selected as a therapeutic option in the early phase of this condition. However, TS patients with vascular liver disorders should be immediately indicated for LT because vascular abnormalities in the liver are likely of congenital origin and distributed throughout the whole liver. In addition, one of the previously reported cases died from respiratory failure and fungal urosepsis, which might be negatively affected by a deteriorated preoperative condition, and therefore delayed LT would have a negative impact with an increased risk for septic complications and failure to thrive [8]. It should be remembered that the TS patients have other organ dysfunctions due to the associated congenital anomalies and related complications. Cardiovascular diseases related to TS are the most serious comorbidities and substantially contribute to the increased mortality rates [17]. In general, significant cardiovascular disease is a relative contraindication to LT. The patients with congenital heart disease have tenuous hemodynamics and may be at a higher risk for hypotension, arrhythmia, and bleeding [18]. Moreover, the potential for air embolism, leading to either pulmonary embolism or paradoxical emboli and cerebral infarction, during the LT procedure and the risk of infective endocarditis needs to be considered [19]. Therefore, cardiovascular diseases, especially congenital heart disease, should be carefully monitored and corrected prior to LT, when feasible, in the TS patients.

## Conclusions

In conclusion, LT is rarely indicated for liver involvement in TS patients; however, patients presenting with severe atrophy and a disturbance of the major portal inflow should be indicated for LT. This strategy is substantiated by the histological findings in the explanted liver of vascular abnormalities, which may be distributed throughout the whole liver to induce marginal liver function.

## Consent

Written informed consent was obtained from the patient for publication of this Case Report and any accompanying images. A copy of the written consent is available for review by the Editor-in-Chief of this journal.

## Abbreviations

FNH, focal nodular hyperplasia; LDLT, living donor liver transplantation; LT, liver transplantation; NRH, nodular regenerative hyperplasia; TS, Turner syndrome

## References

[1] Saenger P (1996). Turner’s syndrome. N Engl J Med.

[2] Roulot D (2013). Liver involvement in Turner syndrome. Liver Int.

[3] Salerno M, Di Maio S, Gasparini N, Rizzo M, Ferri P, Vajro P (1999). Liver abnormalities in Turner syndrome. Eur J Pediatr.

[4] Larizza D, Locatelli M, Vitali L, Viganò C, Calcaterra V, Tinelli C (2000). Serum liver enzymes in Turner syndrome. Eur J Pediatr.

[5] Albareda MM, Gallego A, Enríquez J, Rodríguez JL, Webb SM (1999). Biochemical liver abnormalities in Turner’s syndrome. Eur J Gastroenterol Hepatol.

[6] Gravholt CH, Juul S, Naeraa RW, Hansen J (1998). Morbidity in Turner syndrome. J Clin Epidemiol.

[7] Roulot D, Degott C, Chazouillères O, Oberti F, Calès P, Carbonell N (2004). Vascular involvement of the liver in Turner’s syndrome. Hepatology.

[8] Aucejo F, Ibrahim Z, Hashimoto K, Quintini C, Kelly D, Vogt D (2008). Cruveilhier-Baumgarten disease in a patient with Turner syndrome: case report of a rare indication for liver transplantation. Liver Transpl.

[9] Chintanaboina J, Shah PR, Riley TR 3rd. An unusual occurrence of hepatic granulomas and secondary sitosterolemia in turner syndrome. Case Rep Med. 2015. Epub 2015 Jan 29 10.1155/2015/186718 PMC4326210 25705228

[10] Farrell GC, Larter CZ (2006). Nonalcoholic fatty liver disease: from steatosis to cirrhosis. Hepatology.

[11] Rougier P, Degott C, Rueff B, Benhamou JP (1978). Nodular regenerative hyperplasia of the liver. Report of six cases and review of the literature. Gastroenterology.

[12] Wanless IR (1990). Micronodular transformation (nodular regenerative hyperplasia) of the liver: a report of 64 cases among 2,500 autopsies and a new classification of benign hepatocellular nodules. Hepatology.

[13] Wanless IR, Mawdsley C, Adams R (1985). On the pathogenesis of focal nodular hyperplasia of the liver. Hepatology.

[14] Noe JA, Pittman HC, Burton EM (2006). Congenital absence of the portal vein in a child with Turner syndrome. Pediatr Radiol.

[15] Cramer JW, Bartz PJ, Simpson PM, Zangwill SD (2014). The spectrum of congenital heart disease and outcomes after surgical repair among children with Turner syndrome: a single-center review. Pediatr Cardiol.

[16] Idilman R, De Maria N, Colantoni A, Kugelmas M, Van Thiel DH (2000). Cirrhosis in Turner’s syndrome: case report and literature review. Eur J Gastroenterol Hepatol.

[17] Schoemaker MJ, Swerdlow AJ, Higgins CD, Wright AF, Jacobs PA, United Kingdom Clinical Cytogenetics Group (2008). Mortality in women with turner syndrome in Great Britain: a national cohort study. J Clin Endocrinol Metab.

[18] Asrani SK, Asrani NS, Freese DK, Phillips SD, Warnes CA, Heimbach J, Kamath PS (2012). Congenital heart disease and the liver. Hepatology.

[19] Garbanzo JP, Kasahara M, Egawa H, Ikeda T, Doi H, Sakamoto S (2006). Results of living donor liver transplantation in five children with congenital cardiac malformations requiring cardiac surgery. Pediatr Transplant.

